# Potato (*Solanum tuberosum* L.) Cultivars Interact with Wound Healing Period to Modulate Sprout Emergence, Crop Stand, and Productivity

**DOI:** 10.3390/plants14121830

**Published:** 2025-06-14

**Authors:** Connor L. Buckley, Keegan B. Lloyd, Mohan G. N. Kumar, Jacob M. Blauer

**Affiliations:** 1 Department of Horticulture, Washington State University, Pullman, WA 99164-6414, USA; connor.buckley@wsu.edu; 2 Department of Chemistry and Biochemistry, Brigham Young University, Provo, UT 84602-1030, USA; keeganblloyd@gmail.com

**Keywords:** potato, wound response, wound healing period, superoxide dismutase, peroxidase

## Abstract

The effects of wound healing on crop stand and productivity were examined on the potato (*Solanum tuberosum* L.) cultivars Alturas (Alt), Russet Burbank (RB), and Clearwater Russet (CW). Tuber yields increased linearly with an advancing wound healing period irrespective of the cultivar (R^2^ = 0.91). In contrast to unhealed controls, RB and CW wound-healed for 8 days produced a 6% and 8% greater yield, respectively, while a shorter wound healing period of 2 days increased Alt yield by 7%. Increases in tuber yield, a consequence of enhanced specific tuber weight across wound healing periods, contributed towards increased relative crop value in Alt (13%), RB (22%), and CW (19%). In further lab evaluations, Alt exhibited increased desiccation resistance and was associated with an earlier induction (24 h post-wounding) of feruloyl transferase (FHT) compared to CW and RB. Since FHT facilitates suberin and wax development, delayed FHT induction likely promoted fresh-weight loss in CW and RB compared to Alt. Enzymatic evaluations to assess the production of reactive oxygen species to protect fresh-cut seed found that RB had the highest activities of superoxide dismutase and peroxidase. This study demonstrates the broad benefits of planting wound-healed seed while highlighting opportunities to improve best practices and genetic improvement for wound healing response.

## 1. Introduction

The potato (*Solanum tuberosum* L.) is propagated vegetatively using whole tubers or cut tuber pieces containing at least one lateral bud (eye). Although whole tubers eliminate the need for wound healing, the larger tuber sizes of many commercial cultivars necessitate cutting tubers into eye-containing pieces to reduce the cost of seed by increasing the number of propagules per tuber. The tuber pieces thus prepared are planted with or without wound healing depending on individual grower practices. Wound-healed seed pieces survive and establish a better crop stand, contributing to increased yield [1]. Wound healing forms a hydrophobic barrier impregnated with anti-microbial compounds at the wound site to limit seed rot [2]. Despite the advantages of wound healing, some farmers in the Pacific Northwest (USA) plant ‘unhealed’ seed pieces with variable effects on crop stand and productivity. While some farmers have raised an excellent crop from ‘unhealed’ seed pieces, others have suffered a loss in crop stand as high as 80% [3]. It is possible that such variations in crop stand are likely mediated by cultivar differences in the ability to resist microbial invasion at the wound site and to develop a suberized periderm.

Potato tubers generate reactive oxygen species (ROS) in response to wounding to contain invading pathogens. NADPH Oxidase (NOX) is upregulated first in response to wounding to form anti-microbial superoxide radicals at the wound site. Superoxide dismutase (SOD) then catalyzes the superoxide into hydrogen peroxide, another anti-microbial agent [4,5,6]. The resultant hydrogen peroxide is then used by peroxidases (POX) to incorporate phenolics into the suberin polyphenolic domain (SPP). The sustained production of aromatic and aliphatic components continues as a hydrophobic barrier is produced (suberization) at the wound site. The formation of SPP begins within the first 24 h, while the suberin polyaliphatic (SPA) production begins after 3 days of wounding [2]. Suberization is thus a complex multi-step process mediated by several enzymes to provide components to build the SPP and SPA domains of suberin [4]. The total duration of suberization, and by extent wound healing, is dependent on temperature and genotype, which may take 10–12 days at 12 °C (95% RH) [7]. While several enzymes mediate the formation of the SPA domain of suberin, feruloyl transferase (FHT) plays an important role in the formation of feruloyl esters to support the development of suberin and associated waxes [8,9]. The downregulation of FHT results in the greater loss of fresh weight in tubers [8,10].

Although wound healing is known to protect tubers, grower practices regarding the wound healing period and conditions often vary depending on the cultivar and geographic location (and associated disease risk). While it is generally recommended that growers allow seed tubers to heal before planting, some have successfully produced crops using freshly cut seed, raising questions about the necessity of wound healing. This study examines cultivar-specific differences in wound healing and investigates how the wound healing period influences sprout emergence, crop stand, and tuber yield.

## 2. Results

### 2.1. Evaluation of Wound Healing Ability

**Figure 1:**
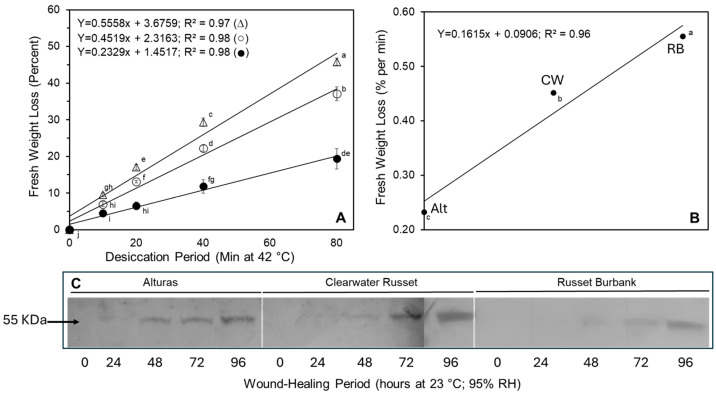
Cultivar differences in the wound healing ability and expression of feruloyl transferase (FHT) in Russet Burbank (RB, ∆), Clearwater Russet (CW, ○), and Alturas (Alt, ●). (**A**) Following wound healing for 96 h (See Section 4), the tuber discs were subjected to forced-air desiccation at 42 °C, and the loss of fresh weight was determined. Irrespective of cultivars, fresh weight declined linearly with the advancing desiccation period. Alt resisted the loss in fresh weight better than CW and RB. The bars on data points signify standard error and differing small letters indicate significance differences (*p* ≤ 0.05). (**B**) The rate of loss in fresh weight was greater in RB than in Alt and CW. (**C**) The immunodetection of FHT (55 KDa) involved in containing desiccation by contributing toward the development of suberin. Each lane was loaded with 38 µg soluble protein. Alt expressed FHT 24 h after wounding, while the expression was delayed until 48 h after wounding for CW and RB. Delayed expression of FHT likely contributed toward the reduced ability to resist desiccation in RB.

Tuber discs from Alturas (Alt), Clearwater Russet (CW), and Russet Burbank (RB) were assessed at 0 (freshly cut), 24, 48, 72, and 96 h after wounding to evaluate their ability to resist fresh-weight loss under forced-air desiccation. While cultivar-specific differences were not apparent until 72 h (see weight loss data in Supplementary File), by 96 h post-wounding, significant differences among cultivars were observed in their resistance to fresh-weight loss (*p* < 0.01). Across all cultivars, a linear decline in fresh weight (R^2^ = 0.99) was observed over an 80 min forced-air desiccation period at 42 °C. Alt displayed the greatest resistance to desiccation, losing only 19% of its fresh weight, compared to 37% for CW and 46% for RB (Figure 1A): a consequence of the highly significant (*p* < 0.001) cultivar by desiccation period interaction on fresh-weight loss. Hence, the percent fresh-weight loss per minute was significantly different for each cultivar (Figure 1B).

### 2.2. Expression of Feruloyl Transferase

The expression of feruloyl transferase (FHT; 55 kDa), an enzyme involved in the synthesis of the hydrophobic suberin barrier, was evaluated via Western blot analysis. FHT was undetectable in freshly cut tuber tissue but was induced in response to wounding. Cultivar-specific differences in FHT expression were evident. Alt exhibited FHT expression as early as 24 h post-wounding, whereas CW and RB did not show detectable levels until after 48 h (Figure 1C).

### 2.3. Superoxide Dismutase and Peroxidase Activities

**Figure 2:**
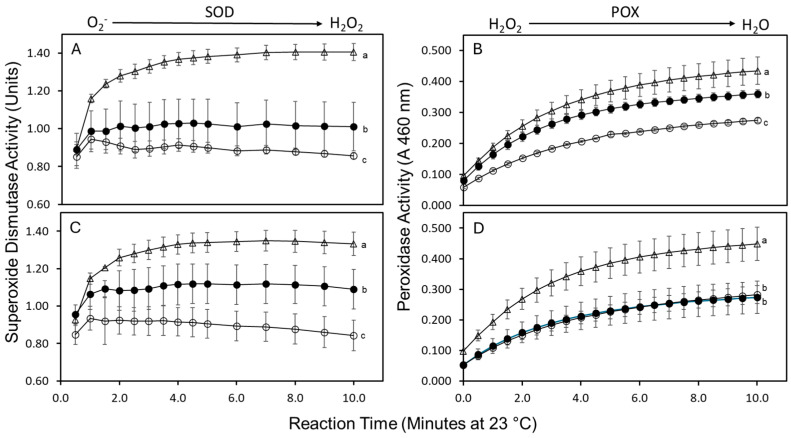
Relative activities of superoxide dismutase (SOD) and peroxidase (POX) on the wounded surface of tubers (Alturas ●, Clearwater Russet ○, and Russet Burbank ∆). Tuber discs (1.7 cm diameter by 2 mm thick) were incubated for 5 min in the buffers for the SOD or POX assay (see Materials and Methods) to collect soluble proteins from the wounded surface. The activities of SOD and POX were determined from the incubation media. The enzyme activities were determined 2.5 h (**A**,**B**) and 5.0 h (**C**,**D**) after wounding. Error bars represent standard error and differing small letters indicate significance differences (*p* ≤ 0.05).

The immediate response of tuber tissue to metabolize anti-microbial reactive oxygen species (ROS) at the wound surface was evaluated by assaying the activities of superoxide dismutase (SOD) and peroxidase (POX). Among the cultivars, RB exhibited the highest SOD activity, exceeding that of Alt and CW by 31% and 47%, respectively. RB maintained elevated SOD activity relative to Alt and CW as early as 2.5 h post-wounding, with this heightened activity persisting at 5 h post-wounding (Figure 2A,C).

A similar trend was observed for POX activity. RB demonstrated the highest POX activity, followed by Alt and then CW (Figure 2B,D). At 2.5 h after wounding, POX activity in Alt and CW was 15% and 38% lower, respectively, compared to RB. At 5 h post-wounding, POX activity in Alt and CW was approximately 40% relative to RB. These findings indicate that RB sustained POX activity at the wound surface, while Alt and CW exhibited a decline in enzymatic activity over time following wounding.

### 2.4. Sprout Emergence and Crop Stand

Sprout emergence was recorded twice (early and late) each year to assess both the rate of early emergence and the final crop stand (see Table 1). The wound healing period significantly influenced early emergence and overall crop stand (*p* < 0.05). In RB and CW, early emergence increased linearly with longer wound healing periods over an 8-day span (2-year average), with R^2^ values of 0.92 and 0.86, respectively. Notably, RB exhibited a 2.7-fold greater early emergence than CW. In contrast, early emergence in Alt followed a quadratic trend, with the highest emergence associated with a 4-day wound healing period (R^2^ = 0.84). Up to 2 days of wound healing, the rate of emergence increased linearly across all cultivars, and differences in percent emergence were not statistically significant (*p* > 0.05).

**Figure 3:**
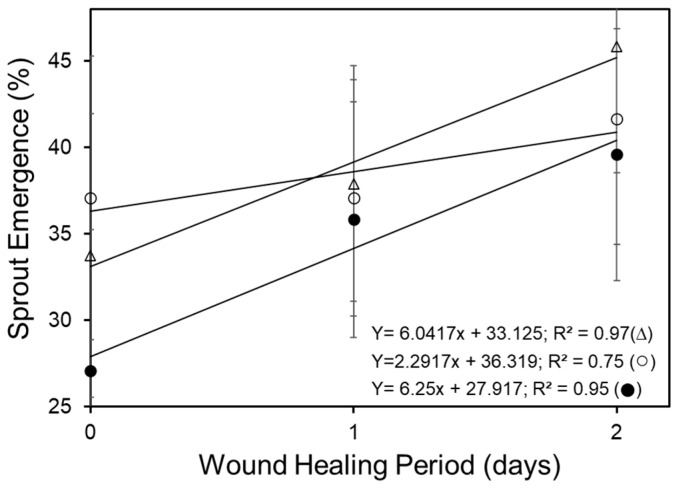
Effect of wound healing period (up to 2 days) on the early (33 DAP) emergence of sprouts (average of 2021 and 2022) for Russet Burbank (∆), Alturas (●), and Clearwater Russet (○). Sprout emergence increased linearly with wound healing period up to 2 days. There were no significant differences between cultivars in terms of emergence after wound healing for 2 days. Bars on data points signify standard error.

Although the overall slopes were similar, the R^2^ values varied notably, indicating differences in how well the linear model explained the variability in each cultivar’s response. Specifically, both Alt and RB showed an approximately 2.6-fold increase in emergence (R^2^ = 0.96) compared to CW (R^2^ = 0.75), indicating the greater consistency and predictability of the response in Alt and RB (Figure 3).

### 2.5. Total and Marketable Yield

Cultivar differences were highly significant for both total and marketable yields (U.S. No. 1 and No. 2 grades; *p* < 0.001; Table 1). The two-year average total yields were 123.7 MT ha^−1^ for Alt, 87.3 MT ha^−1^ for CW, and 74.5 MT ha^−1^ for RB. A significant linear trend was observed across cultivars for the effect of the wound healing period on total yield (WHPLT; *p* < 0.1). Specifically, an 8-day wound healing period resulted in yield increases of 6% in RB and 8% in CW compared to unhealed (fresh-cut) controls.

In Alt, total yield did not increase linearly with longer wound healing periods. Instead, the highest yield (126.9 MT ha^−1^) was achieved with a 2-day healing period. Fresh-cut seed in Alt produced the lowest yield (118.8 MT ha^−1^), marking a 7% reduction. This pattern in Alt paralleled earlier observations for both early and late sprout emergence.

Yields of U.S. No. 1 and No. 2 tubers closely followed trends in total yield, with cultivars having a highly significant main effect (*p* < 0.001). While U.S. No. 1 yields increased linearly with longer wound healing durations (*p* < 0.05), the effect of the wound healing period on U.S. No. 2 yields was not statistically significant.

### 2.6. Tuber Size Distribution, Specific Tuber Weight, and Tuber Count

**Figure 4:**
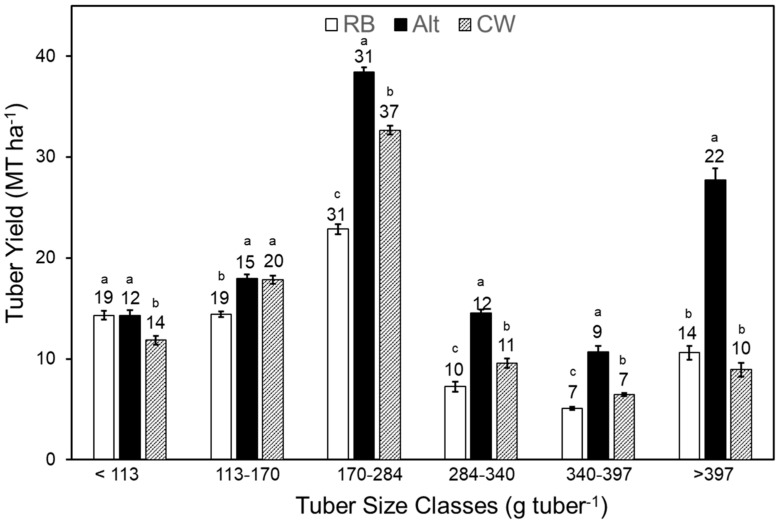
Effect of cultivars on the yield of different tuber size classes and their percent contribution to the total yield (averaged across all wound healing periods). While wound healing period did not affect the yield of different size categories, the differences between cultivars for each individual size category are designated by the letter above the bars (*p* ≤ 0.05). Error bars signify standard error (SE) and the numbers above each bar represent the percent contribution of each category to the total tuber yield (Alt, Alturas; CW, Clearwater; RB, Russet Burbank).

Cultivar selection had a highly significant effect on yield across all tuber size categories, with Alt producing the highest yield in each class (*p* < 0.001; Table 1). The wound healing period also had a significant linear trend effect on the yield of specific tuber size classes: <113 g (*p* < 0.01), 284–340 g (*p* < 0.05), 340–397 g (*p* < 0.05), and >397 g (*p* < 0.1; Table 1), indicating that wound healing generally favoured the production of larger tubers. Across cultivars, the 170–284 g size class dominated the yield profile. In this category, Alt yielded 68% more tubers than RB and 18% more than CW (Figure 4).

**Figure 5:**
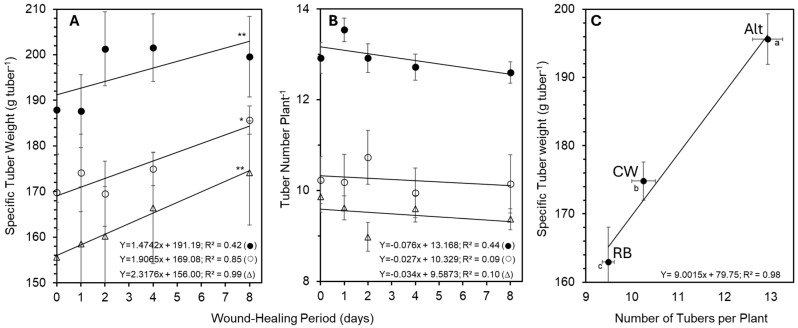
Effect of cultivar and wound healing period on the specific tuber weight (STW) and number of tubers produced per plant (average of 2021 and 2022) in Russet Burbank (∆), Alturas (●), and Clearwater Russet (○). Prior to planting, the seed pieces were wound-healed as described in Section 4. (**A**) Effect of wound healing period on the STW for each cultivar. Asterisk symbols for each line indicate the level of significance of the linear trend of each cultivar for the impact of the wound healing period on STW (** = *p* ≤ 0.05; * = *p* ≤ 0.1). (**B**) Effect of wound healing period on the number of tubers per plant for each cultivar. Note, the linear trend for each cultivar was not significant. (**C**) Correlation between the number of tubers per plant and the STW (averaged over wound healing period; R^2^ = 0.98). Letters indicate significant differences between each cultivar at *p* ≤ 0.05. Bars on data points signify the standard error.

The effect of cultivar on specific tuber weight (STW; index of tuber size) was significant (*p* < 0.05). The average tuber sizes were 196 g for Alt, 175 g for CW, and 163 g for RB. STW increased linearly over the 8-day wound healing period for each cultivar (RB, R^2^ = 0.99; CW, R^2^ = 0.85; Alt, R^2^ = 0.42; Figure 5A). While the linear trend for Alt was significant, the greatest increase in STW occurred with a 2-day wound healing period (R^2^ = 0.86). Extending wound healing from 2 to 8 days did not further increase STW in Alt. In the 2-day wound healing period, Alt produced tubers with 26% and 19% greater STWs than those of RB and CW, respectively.

The main effect of the cultivar on tuber number per plant and per hectare was highly significant (*p* < 0.001), though the wound healing period did not elicit a significant effect on tuber numbers (Figure 5B). Alt produced the most tubers per plant and per hectare (ca. 12.9; 626,619,880), followed by CW (ca. 10.2; 496,282,321) and RB (ca. 9.5; 459,227,592).

Interestingly, a strong positive correlation was observed between tuber number and STW, averaged across wound healing periods (R^2^ = 0.98; Figure 5C), with Alt surpassing both RB and CW in both metrics.

### 2.7. Estimation of Returns

**Figure 6:**
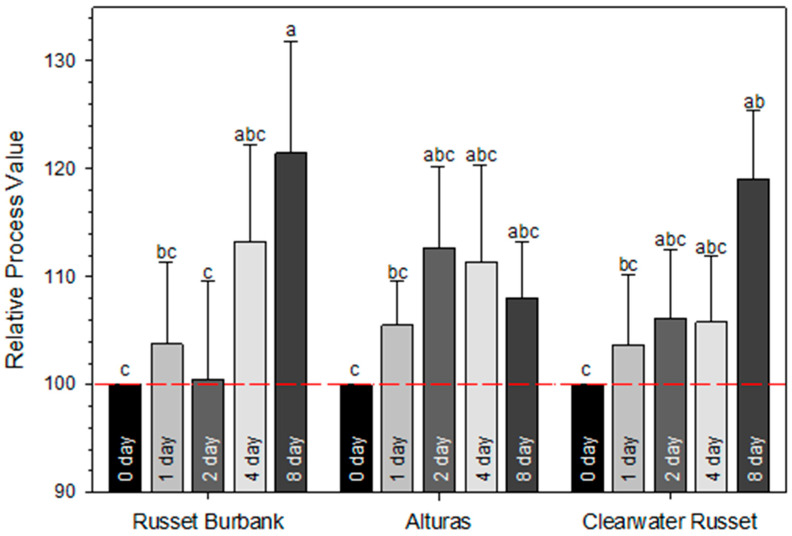
The two-year average relative percent process value for each wound healing period compared to the 0-day (fresh-cut) treatment for each cultivar (Russet Burbank, Alturas, and Clearwater Russet). The red line indicates the 0-day wound healing period set at 100% for comparison. Error bars signify standard error (SE) and letters above the bars indicate differences in means (*p* ≤ 0.05).

Estimated crop values indicated that cultivar selection had a highly significant impact on economic returns (*p* < 0.001; with Alt and CW producing crop values 85% and 29% higher, respectively, than those of RB. To better assess the impact of wound healing period on each cultivar, data was transformed to the ‘relative percent process value’ by calculating the percent crop value for each wound healing period relative to the 0-day (fresh-cut; 100% value) treatment (Figure 6).

The linear trend across cultivars for the effect of wound healing on relative process value was highly significant (R^2^ = 0.96; *p* < 0.001). An 8-day wound healing period produced the highest relative process values for both RB (122%) and CW (119%). In contrast, Alt followed a quadratic trend, with the highest value (113%) occurring at the 2-day wound healing period (R^2^ = 0.85; *p* < 0.1). These economic results closely parallel observed trends in sprout emergence and total tuber yield.

## 3. Discussion

### 3.1. Cultivars Respond Differentially to Wounding

Potatoes are propagated by planting meristem-containing tuber pieces, either with or without wound healing. Although wound healing helps ensure a better crop stand [1] by reducing desiccation and microbial invasion [2,11,12], some growers in the Pacific Northwest (PNW) of the United States have successfully planted freshly cut (unhealed) seed pieces, while others have experienced significant crop losses. Adding further complexity, even among growers who practice wound healing, opinions differ regarding the optimal duration and conditions for the healing period [3]. Consequently, cultivar-specific recommendations for wound healing have yet to be established and developing such guidelines will be valuable to growers.

In the present study, the interaction of cultivar with wound healing period was investigated to establish the impact on fresh-weight loss, sprout emergence, crop stand, tuber yield, and crop value. In vitro evaluations found wound healing ability varied with the cultivar selection (Figure 1). Freshly cut tuber discs had greater fresh-weight loss, regardless of the cultivar, reinforcing the need for a suberized, hydrophobic barrier on the wound surface. Interestingly, cultivar differences in wound healing ability were not evident until 72 h or more after wounding. After 96 h of wound healing, significant differences in fresh-weight loss among the cultivars were evident. Alt was the most efficient in resisting desiccation-induced fresh-weight loss with CW and RB having a 1.9- and 2.4-fold higher rate of fresh-weight loss, respectively. These results demonstrate cultivar-specific differences in wound healing as it relates to the development of the hydrophobic barrier of suberin.

The waxes composing suberin are responsible for retarding moisture loss from the wounded surface [13,14,15,16,17,18,19]. The incorporation of waxes into the suberin matrix is a multi-step process mediated by a number of enzymes [11,20] such as feruloyl transferase (FHT) and fatty acid ω-hydroxylase. Both these enzymes are wound-induced and play a crucial role in wound healing [21,22]. Silencing *CYP86A33* (gene encoding for fatty acid ω-hydroxylase) resulted in a reduction in the aliphatic component of suberin along with a greater loss of moisture from tubers [21]. Similarly, the downregulation of FHT resulted in the reduced incorporation of ferulate and waxes into suberin, and enhanced the permeability characteristics of the resultant wound periderm [8]. While FHT was undetectable in freshly cut tuber tissue as previously reported [21,22], wounding induced its expression in all cultivars. However, the time of expression following wounding differed among cultivars (Figure 1C). In Alt, the induction of FHT occurred as early as 24 h after wounding, while the expression of FHT in CW and RB was evident after 48 h. The cultivar differences in the expression of FHT correlated positively with resistance to fresh-weight loss. The early induction of FHT following wounding likely contributed toward a greater resistance to fresh-weight loss in Alt than in CW and RB (Figure 1A,B).

The development of a wound periderm is a sustained response to wounding and takes several days. An immediate response to wounding involves the production of anti-microbial reactive oxygen species (ROS) on the wound surface [4,23]. The inadequate or delayed formation of ROS on the wounded surface paves the way for microbial infection. In addition, the reduced ability to form ROS affects suberization negatively [24,25]. Thus, cultivar-specific differences in the metabolism of ROS on the wound surface may contribute toward the survival of seed pieces in the field. Thus, the production of ROS on the wound surface was ascertained by indirectly by measuring the activities of superoxide dismutase (SOD; Figure 2A,C) and peroxidase (POX; Figure 2B,D).

SOD and POX are involved in the metabolism of superoxide and hydrogen peroxide, respectively. The SOD and POX activities were detected on the wounded surface, irrespective of the cultivar; however, both SOD and POX activities were higher in RB than Alt and CW. Higher activities of SOD and POX in RB indicated a greater ability to generate superoxide (substrate for SOD) and a greater ability to mediate its dismutation into hydrogen peroxide. It is hypothesized that the improved capability to produce ROS on the wound surface immediately after wounding and the early induction of FHT are ideal for seed-piece survival. While RB demonstrated a greater ability to upregulate ROS immediately following wounding, Alt upregulated FHT expression earlier than RB following wounding and had less fresh-weight loss (Figure 1A–C and Figure 2A–D). Therefore, the development of cultivars capable of rapidly forming ROS, along with an early induction of FHT in response to wounding, will likely benefit seed-piece survival in the field. Such a cultivar would likely produce tubers that would wound-heal rapidly upon harvest and would better resist fresh-weight loss during their postharvest storage life.

### 3.2. Wound Healing Period Affects Emergence and Crop Stand

The wound healing period interacted significantly with each cultivar to affect sprout emergence, total and marketable yields, and tuber numbers per plant, and contributed to differences in tuber size categories, impacting the crop value. The rate of sprout emergence increased linearly with the wound healing period until two days, irrespective of the cultivar. Treatment with a 2-day wound healing period increased the rate of emergence by 2.7-fold in Alt and RB compared to that of CW (Figure 3). An increase in the wound healing period beyond 2 days did not further benefit sprout emergence in Alt, though both RB and CW had linear increases in the rate of early emergence with up to 8 days of wound healing (Table 1). The mechanism(s) by which wound healing affected sprout emergence and crop stand in this study remain speculative, though wound-induced increases in cytokinin levels are known to play a role in sprouting [26,27,28]. Additionally, the cultivar-related differences in the metabolism of ROS and/or the ability to initiate a rapid development of a suberized periderm at the wound site may have further protected against microbial invasion and promoted seed-piece survival [2,7,12].

### 3.3. Cultivars and Wound Healing Period Modulate Tuber Yield

Longer wound healing periods improved total tuber yields across all cultivars. An 8-day wound healing period increased yields by 6% in RB and 8% in CW, corresponding to tuber growth rates of 0.03 and 0.05 MT ha^−1^ day^−1^, respectively. In contrast, a 2-day wound healing period increased total yield in Alt by 7% (0.05 MT ha^−1^ day^−1^). Notably, these increases in tuber yield paralleled the trends observed in early emergence. Given that wound healing has been associated with improved early vigour, crop establishment, and final yield [1], the observed yield increases are likely attributable to the enhanced carbohydrate assimilation enabled by earlier emergence and a longer period of photosynthetic productivity.

Yield improvements were likely driven by changes in average tuber size, tuber count, and tuber size distribution, each contributing to the relative yield of different size classes. Across all cultivars, tubers in the 170–284 g range were predominant, comprising 31–37% of total yield. Alt exhibited the highest yield in this category and produced 2.6- and 3.1-fold more tubers > 397 g than RB and CW, respectively. These larger tubers contributed substantially to Alt’s greater total yield. In contrast, RB produced the lowest overall yield and had a higher proportion of smaller tubers (<113 g). CW yielded 17% more than RB, with 37% of its total yield consisting of tubers in the 170–284 g category.

Generally, tuber number per plant and specific tuber weight (STW) are inversely related due to the partitioning of limited photosynthates among a larger number of tubers. However, Alt achieved the highest total yield by simultaneously producing the greatest number of tubers per plant and the highest STW. Across cultivars, tuber number per plant was positively correlated with STW (Figure 5). Furthermore, STW increased linearly with wound healing duration, indicating that tuber size distribution played a central role in determining total yield. Maximum total yields corresponded with maximum STW, with the largest tubers observed after 8 days of wound healing in RB and CW, and after 2 or more days in Alt. These findings suggest that increases in total yield and market value were primarily mediated through improvements in STW.

### 3.4. Wound Healing Affects Returns

Contracted potato growers generally receive incentives to the base crop price for tubers greater than 170 g. A linear increase in STW with the wound healing period suggests greater returns for planting wound-healed seed over that of freshly cut seed (Figure 6). Both RB and CW responded with a greater relative percent process value from seed that was wound-healed for 8 days (122% and 119%, respectively), while a shorter wound healing period of 2 days generated a 113% relative percent process value for Alt. Thus, the effect of the wound healing period extends beyond seed-piece survival, weight loss, and crop stand, to promote yield and crop value. Studies addressing the ideal cultivar-specific conditions for wound healing (temperature and duration) for different agro-climatic zones will go a long way in improving seed-handling recommendations for growers.

### 3.5. Cost of Wound Healing

Beyond the wound healing interval itself, other important considerations for wound healing cut seed include equipment, operational capacity, and labour costs. The financial, time, and damage costs associated with removing seed tubers from storage for cutting, reloading them into storage under proper wound healing conditions, and then removing them again for shipping must be considered holistically. Variations in equipment efficiency, labour (both skill level and cost), operational capacity (time and equipment availability), climatic conditions (weather and outside temperature), and sanitation practices can significantly influence the overall cost of pre-plant seed wound healing, either positively or negatively.

While these logistical factors introduce variability in the feasibility of wound healing, a quantitative analysis reveals that the financial investment is both measurable and manageable. In the Pacific Northwest (PNW) of the United States, wound-healed seed costs approximately USD 14.76 MT^−1^. With a base seed cost of USD 393.68 MT^−1^ and a planting density of 3.43 MT ha^−1^ (25.4 cm in-row spacing with 81.3 cm furrow spacing and an average seed piece size of 70.9 g), wound healing adds approximately 3.8% to the total seed cost (USD 50.63 ha^−1^). Based on the estimated adjusted gross processing value, the cost of using unhealed seed amounts to 16.0% for RB, 12.6% for CW, and 8.8% for Alt, or 11.7% on average, relative to the contracted payout amount. In contrast, the cost of using wound-healed seed (8 days), with the additional benefits of increased yield and relative percent process value demonstrated herein, would be 14.7% (RB), 11.2% (CW), and 8.6% (Alt), or 10.9% (average) of the contracted payout amount. For Alt specifically, optimizing wound healing to just 2 days could further reduce seed costs by 0.3%, lowering the total to 8.3% of the relative percent process value. On a per-cultivar basis, wound healing would reduce seed costs by 1.3% (RB), 1.4% (CW), and 0.5% (Alt), or 0.8% on average. This percent decrease in seed costs, combined with the additional increase in relative percent process value (13–22%), makes seed wound healing a worthwhile financial investment and highlights the opportunity for developing cultivar-specific recommendations.

Although wound healing has the potential to enhance tuber yields and improve financial returns, it is not without risks. Wound healing involves the repetitive handling and storage of seed tubers, increasing the risk of mechanical damage, bruise, shrink, and microbial invasion. Therefore, decisions regarding the need for and duration of wound healing should be based on several factors: (1) the cultivar’s ability to rapidly generate anti-microbial ROS on the wound surface (as observed in RB), (2) the capacity to quickly develop the wound periderm (as observed in Alt) to limit shrink and microbial infection, (3) the potential to improve financial returns through increased yield and quality sufficient to offset wound healing costs, and/or (4) the grower’s ability to execute effective wound healing practices. Given the variability in cultivar responses, future work should investigate allelic variation within the available germplasm to improve the wound healing potential through marker development and should further refine cultivar-specific recommendations for best wound healing practices.

## 4. Materials and Methods

### 4.1. Plant Material

Certified G3 late-season seed potato (*Solanum tuberosum* L.) tubers (cvs. Alturas, Russet Burbank, and Clearwater Russet) were sourced from a commercial seed-grower in Reardon, WA in January 2021 and October 2022 and stored at 4 °C (95% RH) until planting. Tubers obtained in January 2021 and October 2022 were planted in the month of April for crop years 2021 and 2022, respectively. Cultivars were selected due to their economic value and broad use for potato processing.

### 4.2. Wound Healing and Field Planting

The effect of the wound healing period on crop emergence, crop stand, and tuber yield was examined for two consecutive cropping years (2021 and 2022). Tubers were blocked for apical and basal tuber portion and were cut by hand into 50–64 g seed pieces and wound-healed for 0 (fresh-cut), 1, 2, 4, and 8 days (12 °C, 95% RH). Seed pieces were planted with rows spaced 81.3 cm apart with 28 cm in-row spacing between plants at a depth of 20 cm at the Washington State University Irrigated Research and Extension Unit at Othello, WA (46°47.59200′ N. Lat., 119°02.32200′ W. Long.). The experiment was laid out in a complete randomized block design involving three cultivars interacting with five wound healing periods. The treatments were replicated five times (24 plants per replication). A single plant of the cultivar Chieftain (red-skinned tubers) was grown at each end of the plot to facilitate plot separation at harvest. Each treatment was flanked by a guard row of Russet Burbank to ensure consistent plant-to-plant competition between rows. In-furrow applications of pesticides applied at planting were Quadris^®^ (Syngenta; 593 mL hectare^−1^ of Azoxystrobin; methyl (E)-2-{2-[6-(2-cyanophenoxy) pyrimidin-4-yloxy]phenyl}-3-methoxyacrylate, 22.9% active ingredient) and Admire^®^ Pro (Bayer CropScience LP; 512 mL hectare^−1^ of Imidacloprid; 1-[(6-Chloro-3-pyridinyl)methyl]-N-nitro-2-imidazolidinimine, 42.8% active ingredient). The crop was raised for 147 days (both years) prior to vine-kill using standard agronomic practices for late-maturing russet cultivars in the Columbia Basin of Washington State under centre-pivot irrigation [29]. Soil moisture sensors were used to maintain soil moisture at a minimum of 65% field capacity.

### 4.3. Data Acquisition

Emergence was recorded from May 17th to June 1st in 2021 and from 13 May to 9 June in 2022. Emergence data is averaged for both years as “early” (~33 days after plant; DAP) and as “late” (~45 DAP) emergence for communication purposes in this report. After 147 days, the vines were killed using a flail mower and tubers were allowed to mature for 14 days in the field to facilitate skin set. After the maturation period, individual plots were harvested using a Braco^®^ single-row bagger unit harvester. Individual tubers were washed, weighed, and sorted using a custom-made potato sorter into US No. 1 and 2, and culls, following U.S. grade designations developed by the USDA [30], to estimate the total yield for each field plot.

### 4.4. Financial Value

Estimated financial value for each treatment (e.g., yield and size distribution) was calculated in both 2021 and 2022 following the methods of Pavek et al. [31] and Blauer [32]. Briefly, a mock frozen-processing contract for long-season russet potatoes in the Columbia Basin (WA) was used. Data from individual tuber weights was used to calculate total and marketable yields, including undersize (<113 g). Data was then used to determine financial incentives or penalties for tuber size distribution and corresponding yields. The tuber yield base price was USD 146.61 MT^−1^ and USD 66.14 MT^−1^ for undersize (<113 g). The maximum premium for tubers greater than 170 g was USD 13.23 MT^−1^ with an additional incentive maximum of USD 11.02 MT^−1^ for US No. 1 tubers greater than 170 g. Financial values were transformed to the ‘relative percent process value’ to assess the effects of the wound healing interval on individual cultivar selection with the ‘no wound healing’ (0-day) treatment set to be equal to 100% the crop value for treatment comparisons.

### 4.5. Assessment of Wound Healing Ability

The cultivar differences in wound healing ability were assessed by subjecting wound-healed tuber discs to forced-air desiccation and by using quantified fresh-weight loss as an index of wound healing ability [33]. Tubers were washed free of surface dirt, blocked for size (150–200 g approx.) into four replicates (three tubers per replication), and cut into 2 mm thick slices at the equatorial region. Tuber discs were cut around the perimeter of the tuber slice approximately 2 mm inwards of the periderm using a cork-borer (1.7 cm diameter). The discs were washed free of surface starch with distilled water, dried briefly on paper towels, and placed on a perforated foam mat (Grip-It Shelf and Drawer Liner MSM Industries, Smyrna, TN, USA) laid on moist filter paper in a Petri dish (15 cm diameter). The lids were previously drilled with two holes (1 cm diameter) for ventilation. Tuber discs after 0, 24, 48, 72, and 96 h were frozen in liquid nitrogen and lyophilized for protein extraction (see below). In addition, after 96 h of wound healing, the discs were subjected to forced-air desiccation (42 °C) and the loss of fresh weight was determined gravimetrically at 0, 10, 20, 40, and 80 min intervals during desiccation. The rate of fresh-weight loss was considered to evaluate cultivar differences in wound healing ability.

### 4.6. Superoxide Dismutase and Peroxidase Activities

Cultivar differences in the metabolism of anti-microbial reactive oxygen species (ROS) in response to wounding was determined by assaying the activities of superoxide dismutase (SOD) and peroxidase (POX) at 2.5 and 5.0 h after wounding [34,35,36]. To detect SOD activity, the tuber discs (see above) were incubated in the dark in 1.0 mL of Tris buffer (0.1 M, pH 7.4) containing 13 mM methionine, 0.1 mM EDTA, 2 μM riboflavin, and 150 μM NBT at 23 °C (1.0 mL disc^−1^). Media lacking discs and incubated alongside were used as controls. After 5 min, the discs were discarded and 300 μL of the reaction media was transferred to a 96-well plate (in triplicate). The plate was sealed with an adhesive sealing film (Bio-Rad) and exposed to high-intensity light on an overhead projector. The increase in absorbance at 560 nm was recorded every 2 min for 10 min. SOD activity was expressed as the percentage inhibition of the absorbance of controls by the tuber disc leachate (50% inhibition = 1.0 unit of SOD).

To assay peroxidase activity, the tuber discs were incubated (as above) in 1.0 mL of sodium acetate buffer (0.2 M, pH 5.2) containing 2 mM CaCl_2_ and 150 μM O-dianisidine (Fisher Scientific, Hampton, NH, USA). After 5 min, the discs were discarded, and 275 µL was transferred (in triplicate) to a 96-well plate and the increase in absorbance at 460 nm was monitored for 10 min immediately following the addition of 0.1% (*v*/*v*) hydrogen peroxide (final conc.). POX activity was expressed as the absorbance at 460 nm.

### 4.7. Protein Extraction and Immunodetection

Soluble protein was extracted by mixing 150 mg of ground tissue with 1.5 mL of 0.1 M Tris buffer (pH 6.8) containing 10 mM ascorbic acid and a protease inhibitor cocktail (10 μL per mL; Sigma). The homogenate was mixed by vortexing and centrifuged at 14,000× *g* (10 min; 4 °C). The supernatant was stored at −20 ˚C and used to resolve protein on SDS-PAGE and western analysis. Soluble protein content was determined as per Bradford [37].

Soluble protein (38 µg lane-1) was resolved by denaturing polyacrylamide electrophoresis [38] and transferred to nitrocellulose membrane (0.45 µm), as described previously by Kumar and Knowles [39]. Following transfer, the membrane was blocked (23 °C, 2 h) with PBS-Tween (10 mM sodium phosphate, 150 mM NaCl, and 0.3% Tween-20), and incubated (23 °C, 2 h) with anti-FHT polyclonal primary antibody (diluted 1:7500 with blocking buffer). The detection of FHT bands on the blot involved incubation with alkaline phosphatase-conjugated anti-rabbit secondary antibody (diluted 1:1000 with blocking buffer; Sigma-Aldrich, Burlington, MA, USA) and 100 mM sodium carbonate buffer (pH 9.8) containing 1 mM MgCl_2_, 5.2 µM 5-bromo-4-chloro-indolyl-phosphate (BCIP), and 9.2 µM nitro blue tetrazolium (NBT).

### 4.8. Statistical Analysis

The results of genotype and wound healing period on emergence, tuber yield, and resistance to forced-air desiccation were subject to analyses of variance (ANOVA using JMP 15.2; JMP statistical discovery, LLC; San Francisco, CA, USA). The effect of cultivar, wound healing, and their interaction were separated into single degree-of-freedom contrasts with polynomial trends for the wound healing interval. Mean separation for individual treatments was accomplished using Fisher’s LSD values (*p* < 0.1, 0.05, 0.01, and 0.001). The year-to-year variation was blocked within the ANOVA model and the treatment means were based on the data for two growing seasons.

## 5. Conclusions

The wound healing of potato seed pieces provided measurable benefits across all cultivars studied, including improved sprout emergence, crop stand, tuber yield, and overall crop value. However, the magnitude and nature of these benefits varied significantly by cultivar, indicating that wound healing responses are genetically mediated and require cultivar-specific management to optimize outcomes. For instance, Alturas responded favourably to a shorter, 2-day healing period, while Russet Burbank and Clearwater Russet required an extended 8-day period to maximize yield and economic return. These differences were associated with cultivar-specific variations in suberin biosynthesis, measured though tuber disc desiccation evaluations and through feruloyl transferase (FHT) expression, and by the capacity to generate reactive oxygen species (ROS) at the wound surface to mitigate microbial invasion. While the added cost of wound healing is modest, the average return on investment is potentially substantial, making wound healing a financially sound practice. Given the genetic variation in wound healing capacity and its agronomic implications, future research should focus on identifying genetic markers linked to wound healing traits and developing tailored protocols that maximize cultivar-specific performance under diverse growing conditions.

## Figures and Tables

**Table 1:** Interaction of cultivars with the wound healing period (WHP) affecting sprout emergence, tuber yield, tuber size distribution, and yield of US No. 1 and 2 tubers. Seed pieces were wound-healed as described in the Materials and Methods before planting. (RB, Russet Burbank; Alt, Alturas; CW, Clearwater Russet; MT ha^−1^, metric tons per hectare.)

Cultivar	WHP (days)	Percent Emergence ^a^		Two-Year Average for Yield of Size Classes and Total Tuber Yield (MT ha^−1^)							Tuber Grade ^b^	
		Early	Late	<113 g	113–170 g	170–284 g	284–340 g	340–397 g	>397 g	Total	US #1	US #2
RB	0	34	97	15.9	14.7	22.4	6.1	5.1	9.9	74.2	43.6	14.7
	1	38	95	14.7	15.0	20.9	7.7	5.6	10.1	74.0	45.9	13.4
	2	46	97	13.6	13.2	23.0	6.7	4.5	8.5	69.5	41.8	14.1
	4	48	98	14.2	14.8	23.8	6.6	4.9	12.2	76.5	46.1	16.2
	8	57	100	13.1	14.2	24.2	9.1	5.3	12.5	78.4	48.8	16.5
Alt	0	27	87	14.8	19.2	36.8	14.2	9.2	24.6	118.8	76.3	27.7
	1	36	95	16.3	18.9	39.8	13.9	9.2	25.1	123.3	80.3	26.7
	2	40	93	13.1	18.0	39.1	16.2	11.6	28.9	126.9	84.7	29.1
	4	39	95	13.8	16.7	38.4	14.4	11.1	31.2	125.6	79.9	31.9
	8	33	93	13.5	17.0	37.9	14.0	12.4	28.9	123.7	84.6	25.6
CW	0	37	94	12.8	17.2	31.9	8.0	6.2	8.6	84.7	70.1	1.8
	1	37	97	11.8	18.3	31.7	9.9	5.9	8.2	85.9	72.4	1.7
	2	42	99	13.0	19.4	32.7	9.6	6.7	7.9	89.3	73.8	2.5
	4	42	100	11.5	17.1	32.4	9.2	6.7	8.0	85.0	71.0	2.5
	8	45	98	10.2	17.1	34.6	11.1	6.7	12.0	91.7	79.9	1.6
CV * WHP LSD_0.05_ ^c^		15.4	5.2	3.0	3.0	4.8	2.4	2.6	7.4	10.5	10.3	8.5
CV LSD_0.05_		6.9	2.3	1.3	1.3	2.1	1.1	1.5	3.3	4.7	4.6	3.8
WHP LSD_0.05_		8.9	3.0	1.7	1.7	2.7	1.4	1.1	4.3	6.1	6.1	4.9
CV ^d^		0.05 ^e^	0.001	0.001	0.001	0.001	0.001	0.001	0.001	0.001	0.001	0.001
CVAlt/RB × CW		0.05	0.001	0.05	0.01	0.001	0.001	0.001	0.001	0.001	0.001	0.001
CVRB/CW		ns	ns	0.001	0.001	0.001	0.001	0.05	ns	0.001	0.001	0.001
WHP		0.05	0.05	0.1	ns	ns	0.1	ns	ns	ns	ns	ns
WHP_LT_		0.01	0.01	0.01	ns	ns	0.05	0.05	0.1	0.1	0.05	ns
WHP_QT_		ns	0.1	ns	ns	ns	ns	ns	ns	ns	ns	ns
WHP_CT_		ns	ns	ns	ns	ns	0.1	ns	ns	ns	ns	ns
Alt/RBCW × WHP_LT_		ns	ns	ns	ns	ns	ns	0.05	ns	ns	ns	ns
Alt/RBCW × WHP_QT_		ns	0.1	ns	ns	ns	ns	ns	ns	ns	ns	ns
Alt/RBCW × WHP_CT_		ns	ns	ns	ns	ns	0.1	ns	ns	ns	ns	ns
RB/CW × WHP_QT_		ns	0.1	ns	ns	ns	ns	ns	ns	ns	ns	ns

^a^ Early emergence was measured 17 May 2021 (29 DAP) and 23 May 2022 (37 DAP). Late/final emergence was measured 1 June 2021 (44 DAP) and 3 June 2022 (46 DAP). ^b^ Tuber grades are based on the standards from the United States Department of Agriculture for potato grading for quality and are designated as either a US Number 1 or US Number 2. ^c^ LSD_0.05_, Least Significant Difference at *p* < 0.05. The LSD value is given for the cultivar, the wound healing period, and the interaction. ^d^ Sources of variation for single degree-of-freedom (df) planned comparisons (LT, linear trend; QT, quadratic trend; CT, cubic trend; Dev, deviations). ^e^ *p* values for the single df contrasts (ns, not significant). Note: Only those contrasts that significantly affected one or more of the agronomic variables are shown.

## Data Availability

Data are contained within the article and Supplementary Materials.

## Supplementary Materials

The following supporting information can be downloaded at: https://www.mdpi.com/article/10.3390/plants14121830/s1, weight loss dataset.

## Abbreviations

The following abbreviations are used in this manuscript:

Alt	Alturas
CW	Clearwater Russet
RB	Russet Burbank
DAP	Days after planting
FHT	Feruloyl transferase
USA	United States of America
PNW	Pacific Northwest
ROS	Reactive oxygen species
NOX	NADPH Oxidase
SOD	Superoxide dismutase
POX	Peroxidases
SPP	Suberin polyphenolic domain
SPA	Suberin polyaliphatic domain
RH	Relative humidity
G3	Generation 3
USDA	United States Department of Agriculture
US No. 1	United States Number 1
US No. 2	United States Number 2
WA	Washington
MT	Metric tons
ha	Hectare(s)
USD	United States Dollars
NBT	Nitro blue tetrazolium
BCIP	5-bromo-4-chloro-indolyl-phosphate
ANOVA	Analysis of variance
STW	Specific tuber weight
